# Commentary The Importance of Cost Estimation for Molecular Epidemiology Studies

**DOI:** 10.2188/jea.JE20160078

**Published:** 2016-10-05

**Authors:** Carlo La Vecchia

**Affiliations:** Università degli Studi di Milano, Milano, Italy

**Keywords:** molecular epidemiology, cost, study design

In the current issue of the *Journal of Epidemiology*, Mishiro et al^1^ present a very careful and detailed cost estimation for a molecular epidemiology cohort study in a model region of Japan. Their approach and key points can be extended to other studies worldwide in order to rationalize planning and costs of molecular epidemiology cohort studies. Of particular interest—though subject to a range of errors due to unexpected events—is their careful allocation and definition of time requested for each unit of personnel, since personnel costs account for the major proportion of the total costs of epidemiological studies.

Such a detailed definition of costs may well become a benchmark for other epidemiology studies worldwide. There are two aspects, however, that require additional attention. First, the new cohort described by Mishiro et al,^1^ which includes 7400 subjects, is relatively small for the subsequent analysis of most of its possible outcomes, despite its comprehensive and innovative data collection compared to cohorts defined in the 1980s and 1990s.^2^^,^^3^ Thus, this dataset will inevitably need to be integrated with data from other cohort studies from Japan and other areas of the world; a similar project was recently developed within a network of cohort studies that included 23 European and 3 non-European studies (the Consortium of Health and Ageing), which includes over 680 000 elderly.^4^ The same line of reasoning applies to the potential integration of some data from this project for the design of nested case-control studies in related consortia.^5^ Such integration has to be considered in the study design and planning phases and may have some—though partial and modest—influence on definition of cost estimations.

An additional issue in planning a molecular epidemiology cohort study is the definition of the biological material to be stocked and subsequently analyzed, as well as the modalities of its stocking, in order to assure greater and optimal utilization in the long term, an element which may not necessarily be easy to define in advance. This correlates with the definition of epidemiological information to be collected, which again has implications for study costs.

In addition, starting from planning and cost estimation of a cohort study, the burden of its follow-up should be considered in advance as an important future cost element. Record linkage across several databases now offers important potentialities for optimizing follow-up at low cost,^6^^,^^7^ but this may not cover the practical totality of subjects to be followed (ie, generally over 95% of the original cohort). Consequently, active follow-up may be required,^8^ and this may appreciably influence subsequent costs of the study. Thus, the burden of follow-up should also be considered and optimized in study planning.

Despite these limitations, the paper by Mishiro et al^1^ constitutes an important exercise towards cost estimation of a baseline survey for a cohort study that includes biological and molecular information. Therefore, this manuscript should be regarded as an example for practical definition and methodology optimization of future studies in other populations.

Carlo La Vecchia
Department of Clinical Sciences and Community Health
Università degli Studi di Milano, Milano, Italy
